# Cardiac MRI with concurrent physiological monitoring using MRI-compatible 12-lead ECG

**DOI:** 10.1186/1532-429X-14-S1-P231

**Published:** 2012-02-01

**Authors:** Zion Tse, Charles Dumoulin, Gari Clifford, Julien Oster, Michael Jerosch-Herold, Raymond Kwong, William Stevenson, Ehud J Schmidt

**Affiliations:** 1 Brigham and Women's Hospital, Harvard Medical School, Boston, MA, USA; 2 University of Cincinnati College of Medicine, Cincinnati, OH, USA; 3 University of Oxford, Oxford, UK

## Background

High fidelity 12-lead ECG is important for physiological monitoring during cardiovascular interventions. A dominant ECG R-wave is essential for synchronizing cardiac MRI. Obtaining the real ECG in MRI is challenging due to a superimposed Magneto-Hydro-Dynamic (MHD) voltage (VMHD) [1], & strong induced voltages from MRI switched gradients. Detecting acute ischemia by S-T segment [2] is difficult due to VMHD peaks that occur during this period. We previously [1] presented (A) an adaptive MHD filtering procedure, based on 3 ECG training sets, & (B) R-wave detection based on 3-D ECG multichannel analysis. We extended our solution with (C) an electronic switching circuitry that blocks ECG transmission during Gradient Ramps and Radio-Frequency Transmission (GR&RF), providing; (1) 12-lead diagnostic-quality ECG free of MHD & GR&RF, (2) beat-to-beat stroke volumes (SV) estimated from VMHD, (3) accurately-gated cardiac MR images.

## Methods

**Figure 1:**
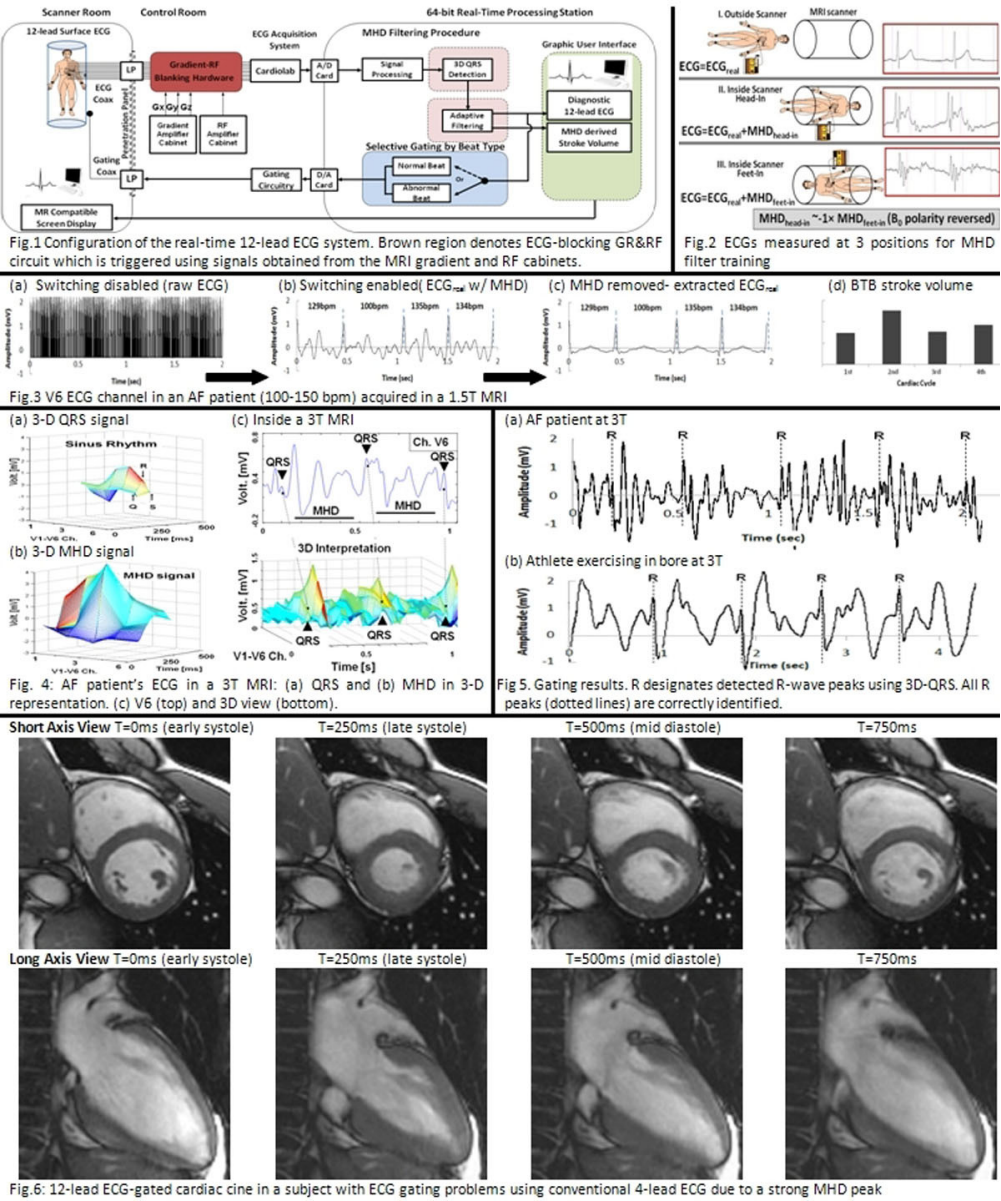


MR-compatible 12-lead ECG system (Fig.1) has 10 leads attached to patient, which transmit ECGs to the penetration panel on coaxial cables equipped with ferrite filters. Outside the room, an electronic circuit prevents ECGs from reaching the GE Cardiolab-IT digital ECG-recording system during GR&RF periods. Cardiolab streams out high-fidelity ECGs to a 64-bit computer, in which real-time MHD filtering & QRS detection are implemented. System outputs are real ECG, SV & R-wave triggers for scanner gating. System was validated on; 2 Atrial Fibrillation (AF), 1 Premature Ventricular Contraction patient, & 5 healthy subjects, including an exercising athlete. Imaging was performed at 1.5T & 3T, with 12-lead ECG quality and electrode temperatures recorded. Before imaging, 20-sec breath-held ECGs were taken at 3 positions for MHD filter training (Fig.1 (2)). After imaging, the derived real ECGs were compared to ECGs measured periodically outside MRI for validation. Fig.1 (4) shows an improved R-wave detection using a 3-D ECG representation, consisting of a time, a voltage axes & a channels axis (ECG channels V1-V6).

## Results

AF ECG processing during a GRE scan (Fig.1) (3): (a) Raw ECG V6 is dominated by GR&RF noise, which is removed (b) by the switching circuit, leaving real ECG + VMHD. VMHD is removed (c) using adaptive filters & QRS detection, also providing (d) SV estimation (irregular due to changes in ventricular-filling). In (c) S-T segment is preserved for ischemia monitoring. 3-D QRS R-wave detection is demonstrated (Fig.1) (4): (a) Sinus rhythm 3-D QRS shape is distinguishable from (b) 3D MHD shape, (c) even when MHD voltage dominates. 3D-QRS (Fig.1(5)) correctly detected the QRS in ECGs acquired at 1.5T & 3T in all 8 subjects. <5msec computational speed enabled accurate MRI triggering, permitting cine MRI in subjects (Fig.1 (6)) where 4-lead ECG failed due to a strong MHD peak.

## Conclusions

The 12-lead ECG system acquired ECGs without MHD & GR&RF artifacts, preserving the S-T segment for ischemia monitoring, allowing SV estimation and robust ECG-gated cardiac MRI.
